# 

**Published:** 1886-04-20

**Authors:** 


### TABLE OF CONTENTS.

ORIGINAL AND SELECTED ARTICLES.

Tetanus, by H F. Scott, M.D., of Atlanta, Ga...121
Treatment of Nevus...126
The Cure of Carbuncle with Caustic and Strips, by J. McF Gaston, M D., Atlanta, Ga...127
A Foreign Body in the Eyeball Removed by Means of a Magnet, by A. G. Hobbs, M. D., Atlanta, Ga...129
On Tapping the Pericardium, by Prof. T. G. Stewart, M. D, Edinburgh, Scotland...130
Jacke's of Sodium Cilicate in the Treatment of Spinal Disease, by Dr. S. T. De La Mater, of Fayetteville, N. Y...132
Diagnosis of Infantile Diseases, by Benjamin A. Bradley, M.D, Cincinnati, Ohio...134
The Administration of Urethane...135
Calcium Hippurate in Dyspesia, Diabetis, etc.; Granulations...136

ABSTRACTS AND GLEANINGS.

Naphthalin in Diarrhœa and Tpphoid Fever...137
Carbolic Acid for Phthisis; Dr. K I. Graves...138
Treatment of Pneumonia; For Dyspepsia...139
Infection by Catgut Sutures; Mother's Mark; Strength of Carbolic Acid Solution for Injection into Hemorrhoids...140
Induction of Premature Labor; A Strong Argument in Favor of Vaccination...141
Stomatitis Materna, or Nurses Sore Mouth; Opium in Infantile Diarrhœa; The Pharmaceutical and Therapeutic Use of Algin...142
Placenta Prævia; The Use of Antipyrine During Pregnancy; For Inflamed Prostate...143
Phenic Acid in Intermittent Fever; Atropine to Prevent Shock after Operation; Pilocarpin in Pneumonia...144
Use of Sulphur in the Treatment of Chronic Dysentery; Enuresis Nocturna; Water for Infants...145
A New National Association of Physicians; Manaca in Muscular Rheumatism; Tests for Albumen...146
The Interval between Marriage; Sulphate of Aniline for Epilepsy; Lanolin, or Wool Fat; Bee-stings...147
Treatment of Acute Tonsillitis; Vomiting in Pregnancy; Granular Lids; To Reduce Hernia...148
Vaginal Pastiles; Chronic Enlargement of Tonsils; Paraldehyde as an Antidote to Strychnine; Prevention of Cancer...149
Tsuchiakabi; Action of Coff e on Pruritus; A Little Girl; Salicylate of Lithia in Rheumatism; Quinine in Irritable Ltomach...150

SCIENTIFIC ITEMS.

Balata; Sight for the Blind; Galvanized Corpses; Our Disappearing Forests...151
The Fuel Problem; Hearing of Infants...152

PRACTICAL NOTES AND FORMULÆ.

The Treatment of Cholera Infantum; Menthol Disks for Sick Headache; Bichloride of Mercury in Phthisis; Typhoid Fever...153
Irritability of the B adder; Croup; Ague; Amenorrhœa...154
Brown's Iron Bitters; Thompson's Eye Water; Resorcin in the Treatment of Gonorrhœa; Catarrh; To Prevent Pitting in Small-Pox; For Removing Warts...155

EDITORIALS AND MISCELLANEOUS.

Editorial Notices...156
International Medical Congress...157
Publishing the Transactions of the Georgia Medical Association...158
Where is the Responsibility...161
Books and Pamphlets Received; New Advertisements; Special Notices...164

